# Phenotypes in Children With *SYNGAP1* Encephalopathy in China

**DOI:** 10.3389/fnins.2021.761473

**Published:** 2021-12-02

**Authors:** Huiting Zhang, Liu Yang, Jing Duan, Qi Zeng, Li Chen, Yu Fang, Junjie Hu, Dezhi Cao, Jianxiang Liao

**Affiliations:** ^1^Shenzhen Children’s Hospital, China Medical University, Shenzhen, China; ^2^Guangdong Women and Children Hospital, Guangzhou, China; ^3^Department of Neurology, Shenzhen Children’s Hospital, Shenzhen, China; ^4^Shenzhen Children’s Hospital, Shantou University, Shenzhen, China

**Keywords:** *SYNGAP1* gene, intellectual disability, China, epilepsy, neurodevelopmental disorder

## Abstract

**Objective:** We aimed to explore the associated clinical phenotype and the natural history of patients with *SYNGAP1* gene variations during early childhood and to identify their genotype–phenotype correlations.

**Methods:** This study used a cohort of 13 patients with epilepsy and developmental disorder due to *SYNGAP1* mutations, namely, 7 patients from Shenzhen Children’s Hospital between September 2014 and January 2020 and 6 patients from previously published studies. Their clinical data were studied.

**Results:** A total of 13 children with *SYNGAP1* gene variants (eight boys and five girls) were identified. The age of disease onset was in infancy. Mutations were located between exons 8 and 15; most were frameshift or truncated mutations. Four mutation sites (c.924G > A, c.1532-2_1532del, c.1747_1755dup, and c.1735_1738del) had not been reported before. All patients had global developmental delay within the first year of life, and intellectual impairment became gradually apparent. Some of them developed behavioral problems. The developmental delay occurred before the onset of seizures. All seven patients in our cohort presented with epilepsy; myoclonic seizures, absence seizures, and epileptic spasms were the most common seizure types. Abnormal electroencephalograms were identified from five patients before the onset of their seizures. All patients suffered from drug-resistance seizures. However, comorbidities such as behavioral problems were less frequently observed.

**Conclusion:** The most common age of disease onset in *SYNGAP1* gene mutations is in infancy, while neurodevelopmental delay and epilepsy are the major phenotypes. They have a higher percentage of drug-resistant epilepsy and epileptic spasms than those in previous reports. We should give attention to the patients with abnormal EEGs without seizures and think about the suitable time of the anti-seizure medications for them. We have not found the genotype–phenotype correlation.

**Trial registration:** Chinese Clinical Trial Registry, Registration number: ChiCTR2100049289 (https://www.chictr.org.cn/listbycreater.aspx).

## Background

Previous studies have shown that the brain-specific synaptic guanosine triphosphatase (GTPase) activating protein (SynGAP) is important for synaptic plasticity. The synaptic Ras GTPase activating protein 1 (*SYNGAP1*) gene (MIM:603384) is located on chromosome 6p21.3. The protein encoded is SynGAP, including Ras and Rap GTPases. The protein is enriched in the postsynaptic membranes and plays an essential role in the plasticity of synapses that excite them (Gamache et al., 2020). The mutation of the *SYNGAP1* gene can lead to an imbalance of excitement and inhibition in the central nervous system, which affects the formation of synapses during learning and memorizing (Nakajima et al., 2019; Araki et al., 2020). SynGAP sequence contained six various predicted functional domains: pleckstrin homology (PH) domain (27-253AA), C2 domain (263-362AA), RasGAP domain (392-729AA), SH3 domain (785-815AA), coiled-coil (CC) domain (1189-1262AA), and other C-terminal domains of unknown function (Hamdan et al., 2009; Berryer et al., 2013; Gamache et al., 2020). The SynGAP protein has multiple biological functions and interacts with numerous proteins (Meili et al., 2021). The loss of function of the *SYNGAP1* gene has been linked to a variety of neurodevelopmental disorders (NDD), including autism spectrum disorder (ASD), intellectual disability (ID), and epilepsy. Encephalopathy caused by the variation of the *SYNGAP1* gene has also been referred to as mental retardation type 5 (MRD5) (Weldon et al., 2018; Agarwal et al., 2019). *SYNGAP1* variants accounted for 0.75% of all NDD (Deciphering Developmental Disorders Study, 2015). MRD5 has a reported incidence of one to four people in 10,000. It is involved in about 0.5–1.0% of all ID patients (Mignot et al., 2016). MRD5 is believed to be the leading cause of ID.

*SYNGAP1* encephalopathy is a rare disease with approximately 200 reported cases worldwide (Agarwal et al., 2019). Most of these patients are from Europe and only six patients have previously been described in detail from China; hence, the clinical and genetic characteristics regarding Chinese patients with mutation of the *SYNGAP1* gene were scarce. In this study, we additionally identified seven patients with *SYNGAP1* gene mutations. We reviewed the phenotypes and molecular structures of these patients in details together with those previously reported in China and analyzed the genotype-phenotype correlation. We reported four novel mutations of the *SYNGAP1* gene.

## Materials and Methods

### Patients

We retrospectively analyzed the clinical data of seven patients who had been diagnosed with developmental disorder comorbid epilepsy from September 2014 to January 2020 at Shenzhen Children’s Hospital. This study was approved by the Institutional Review Board of Shenzhen Children’s Hospital. Written informed consents were obtained from the patients’ parents or legal guardians for the participation of this study and publication of data.

### Inclusion and Exclusion Criteria

Children under 18 years of age were recruited in this study. The results of their genetic testing suggested deleterious *SYNGAP1* variants, and the variants were classified as “likely pathogenic” or “pathogenic” according to the American College of Medical Genetics and Genomics (ACMG) guideline. Patients with epilepsy caused by intracranial infection, congenital metabolic disease, head trauma, structural abnormality of the brain, and birth asphyxia were excluded. Patients with other genetic mutations or chromosomal abnormalities were also excluded.

### Gene Sequencing and Validation Analysis

Genetic tests were carried out on all recruited children. *SYNGAP1* gene mutations were identified in seven children using trio-based whole-exome sequencing (WES). The test was verified for their probands. The pathogenicity of the variants was assessed according to the ACMG guidelines (Richards et al., 2015).

### Literature Review of Previously Published Reports

We used the search terms “China,” “*SYNGAP1*,” and “mutation” in the China Knowledge Network (in Chinese), Wanfang Data (in Chinese), and PubMed. We also compared the mutation sites of the present study with the database.

## Results

### Demographic Information

Seven patients (five males and two females) were recruited in this study. Six patients were of Han ethnicity and one patient was of Tujia ethnicity (Patient 2). The children were aged 31 to 75 months (median, 57 months) when reported. All patients reported no related family history related to developmental disorder and epilepsy, and the birth history was unremarkable. In addition, we collected data from another six patients from previously published literature.

### Clinical Symptoms

All patients showed varying degrees of growth and developmental retardation and intellectual disability within the first year of life (Tables 1, 2). The ability to speak was severely impaired. One patient remained verbally disabled until the last follow-up at 31 months. The mean age of the first spoken word for the remaining six patients was 34 months (median age was 37 months, range between 18 and 55 months). Motor developments, including head control, sitting, standing, and walking, were delayed but better than language development. Two patients had sleep problems, manifested as the difficulties of falling asleep and waking up at night. One presented with aggressive behavior (Table 1). Severe, moderate, and mild developmental delay was noted in one, five, and one patient, respectively. In total, two patients older than 3 years old were diagnosed with ASD, both of whom had moderate developmental delay. Feeble verbal and non-verbal communication skills and impaired social interactions were observed in patients with ASD.

**TABLE 1:** Clinical information of seven patients in our cohort.

ID	1	2	3	4	5	6	7
Gender	Female	Male	Male	Male	Male	Female	Male
Age (months)	75	69	60	59	60	46	31
Developmental delay	Moderate (32 months)	Severe (54 months)	Moderate (38 months)	Moderate (40 months)	Mild (32 months)	Moderate (29 months)	Moderate (20 months)
Age of walking (months)	17	24	18	28	19	18	20
Age of standing (months)	15	18	/	18	/	11	18
First word spoken (months)	44	18	55	30	18	44	Verbal disability
Developmental regression	−	−	−	−	−	−	+
ASD	+	−	+	−	−	−	−
Sleeping problem	−	−	+	−	−	−	+
Seizures	+	+	+	+	+	+	+
Neuroimaging	+	−	−	−	+	−	−
Metabolic screening	−	−	−	−	−	−	−

**TABLE 2:** Clinical information of six patients reported previously.

ID	8	9	10	11	12	13
Gender	Male	Male	Female	Female	Female	Male
Age (months)	96	69	9	34	46	48
Developmental delay	Moderate (96 months)	Mild (42 months)	Mild (9 months)	+	Profound	+
ASD	+	−	/	/	/	+
Seizures	+	+	+	+	−	−
Neuroimaging	−	−	−	+	+	−
Metabolic screening	−	−	−	/	−	−

All the patients with *SYNGAP1* gene mutations had seizures and these patients were prescribed anti-seizure medications (ASMs) (Table 3). The age of onset of the seizure ranged from 0 to 4 years, while the mean age at diagnosis was 26.8 months (median age 30 months). Types of seizures during the course of disease consisted of myoclonic seizures, myoclonic absence, or absence seizures, and epileptic spasms. Four children had myoclonic seizures, two of which had falling seizures. Three children had eyelid myoclonia, and two of them were together with absence. Three patients had epileptic spasms. The triggers of seizures have been identified in five patients. Eating-induced seizures were observed in 42.8% (three of seven) of patients, and no light-evoked seizures were observed in our cohort. None of the patients had status epilepticus. Developmental regression occurred in two individuals after the onset of seizures. We also found no significant features in the physical or neurological examinations except for one patient who developed acquired microcephaly (Patient 1). In addition, the summary of the reported data of the six children is shown in Table 2. All 13 patients suffering from mild to severe developmental delay and seizures were observed in 11 of the 13 patients.

**TABLE 3:** Epilepsy characteristics of seven patients.

ID	1	2	3	4	5	6	7
Age of seizure onset	37 months	30 months	30 months	10 months	26 months	25 months	30 months
Seizure type	Myoclonic seizures, absence, falling seizures	Eyelid myoclonia, myoclonic seizures with eyelid myoclonic	Myoclonic seizures, West syndrome	Eyelid myoclonia with atypical absences	Eyelid myoclonia, falling seizures	Absence with eyelid myoclonia, eyelid myoclonia	Myoclonic seizures
Precipitating factors	Weariness	−	Eating (suspected)	−	Eating	Eating	Crying
Status epilepticus	−	−	−	−	−	−	−
Seizure frequency	6–7/day	10 + /day	20 + /day	7–8/day	7–8/day	6–7/day	10–15/day
Alterations of seizure type	−	+	+	+	−	−	+
ASMs	LEV-TPM-VPA	LEV-VPA-ZNS	LEV-VPA-CZP-ACTH-prednisone	VPA-LEV-NZP	VPA-LEV-LCM	OXC-VPA (OXC discontinued) -LEV-NZP-CLB-LTG-KDT	VPA-NZP-LEV
Seizure free	+	+	+	−	−	−	−
Seizures at last follow-up	Seizure-free for 24 months	Seizure-free for 14 months	Seizure-free for 18 months	Seizure-free for 10 months	Eyelid myoclonia	Absence with eyelid myoclonia	Myoclonus
EEG before seizure onset	Abnormal	Abnormal	Normal	Abnormal	/	Abnormal	Abnormal
EEG, generalized spike and waves	+	+	+	−	+	+	+
EEG, multi-focal spike and waves	+	+	+	+	+	+	+
EEG, hypsarrhythmia.	−	+	+	−	−	−	+
Video-EEG Captured seizures	Myoclonic seizures with absence.	Myoclonic seizures, eyelid myoclonia, epileptic spasms.	Epileptic spasms.	Eyelid myoclonia.	Epileptic spasms, myoclonic seizures.	Atypical absence, eyelid myoclonia with absence, absence atonic seizures, focal seizures	Epileptic spasms.

*VPA, valproate; ZNS, zonisamide; LEV, levetiracetam; NZP, nitrazepam; CZP, clonazepam; CLB, clobazam; OXC, oxcarbazepine; LTG, lamotrigine; TPM, topiramate; ACTH, adrenocorticotropic hormone; KDT, ketogenic diet therapy; LCM, 1acosamide.*

### Gene

**FIGURE 1:**
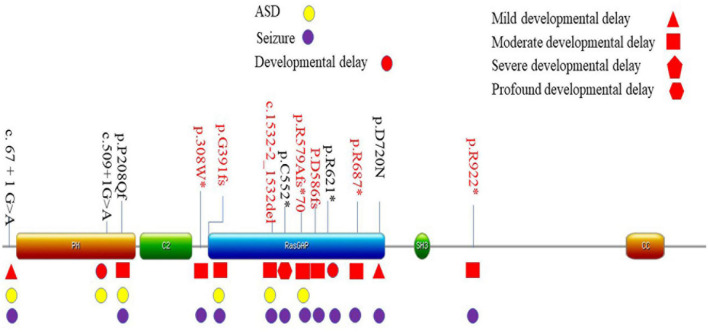
Location of mutation sites of 13 patients in China. Localization of the *SYNGAP1* mutations identified in Amino acid positions are based on the longest SynGAP isoform (NM_006772.3). The main phenotype was listed in corresponding domains. *These variations caused truncated proteins.

Seven different pathogenic mutation sites were detected in this study, and seven patients were confirmed as *de novo* mutations. Three sites [c.2059C > T (Vlaskamp et al., 2019), c.2764C > T (Parker et al., 2015), and c.1167_1168del (Yang et al., 2014)] had been previously reported. The other four loci had not been reported. A total of 13 mutation sites have been identified in Chinese children, including 6 mutations that had been observed in previously reported cases (Lu et al., 2019; Gao et al., 2020; Tian et al., 2020). The mutation sites of the 13 patients were located in exons 1, 5–8, 10–13, and 15 (Supplementary Figure 1). The mutation sites of the 13 children on the domains of SynGAP are shown in Figure 1. Among the mutations of the seven patients recruited in this study, three had frameshifted mutations, one patient had splice mutation, and three patients had non-sense mutations. These variations caused truncated proteins in six patients and aberrant splicing proteins in one patient (Table 4). We found that the mutations distributed PH to RasGAP domain of SynGAP. Of 13 variations, 7 were located on the RasGAP domain, 2 were on the PH domain, and 4 others were on the DUF domains.

**TABLE 4:** List of 13 patients with *SYNGAP1* gene molecular information in China.

ID	cDNA	Mutation type	Changes in protein	ACMG	Pathogenicity	Inheritance
1	c.1735_1738del	Frameshift	p.R579Afs*70	PVS1 + PS2 + PM2	Pathogenic	*De novo*
2	c.924G > A	Non-sense	p.W308*	PVS1 + PS2 + PM2	Pathogenic	*De novo*
3	c.1167_1168del	Frameshift	p.G391fs*27	PVS1 + PS2 + PM2	Pathogenic	*De novo*
4	c.2059C > T	Non-sense	p.R687*	PVS1 + PM6 + PM2 + PP4	Pathogenic	*De novo*
5	c.1747_1755dup	Frameshift	p.D586fs	PVS1 + PM6 PM2	Pathogenic	*De novo*
6	c.2764C > T	Non-sense	p.R922*	PVS1 + PS1 + PS2 + PM4	Pathogenic	*De novo*
7	c.1532-2_1532del	Splice site	/	PVS1 + PM2 + PM6	Pathogenic	*De novo*
8	c.623delC (Tian et al., 2020)	Frameshift	p.P208Qfs*15	PVS1 + PM2 + PM6	Pathogenic	*De novo*
9	c.67 + 1G > A (Tian et al., 2020)	Splice site	/	PVS1 + PM2 + PM6	Pathogenic	*De novo*
10	c.2158 G > A (Tian et al., 2020)	Missense	p.D720N	PM2 + PM6 + PP3 + PP4	Likely pathogenic	*De novo*
11	c.1861C > T (Gao et al., 2020)	Non-sense	p.R621*	PVS1 + PS2 + PM2 + PP4	Pathogenic	*De novo*
12	c.1656C > A (Lu et al., 2019)	Non-sense	p.C552*	PVS1 + PM2 + PM6	Pathogenic	*De novo*
13	c.509 + 1G > A (Pei et al., 2018)	Splice site	/	PVS1 + PM2 + PM6	Pathogenic	*De novo*

*These variations caused truncated proteins.

### Electroencephalogram Findings, Imaging Results, and Metabolic Screening

Screening tests of metabolic diseases in all seven children were unremarkable. Patients 1 and 6 showed mild demyelination and widened extracerebral interspace in the left temporal polar, respectively, in the brain magnetic resonance imaging (MRI). No obvious abnormality was found in the brain MRI or computed tomography (CT) from the remaining five patients. Notably, abnormal electroencephalograms (EEGs) were identified in five patients before seizure was diagnosed. The EEG was unremarkable in one patient. Another patient (Patient 5) had not obtained EEG before the onset of the seizures. All of our patients underwent video EEG at least once for 12 h. Six patients had a diffuse slow background rhythm, and no predominant background was found in one patient. Six patients had generalized spikes and waves, single or repetitive. All patients had multi-focal spikes and waves on EEG. Three in seven children had hypsarrhythmia. Clinically, they had epileptic spasms, and two of them had developmental regression. Clinical seizures were captured by video EEG in all children. Eyelid myoclonia with absence in three patients was reported, epileptic spasms were observed in three, myoclonic seizures in two, atypical absence in one, myoclonic seizures with absence in one, absence with atonic seizures in one, and focal seizures in one. Because our patients were young, only one patient finished the test of hyperventilation and photic stimulation, and the result was negative.

### Treatment Outcome of Seizures

All patients were receiving ASM treatments and rehabilitation training. Three became seizure-free for more than 1 year after 6 months of anti-seizure therapy. Patient 1 was seizure-free for 2 years with a medication regimen of sodium valproate combined with topiramate and levetiracetam. Patient 2 was seizure-free for 14 months with a medication regimen of sodium valproate combined with levetiracetam and zonisamide. Patient 3 experienced recurrence after 18 months of seizure-free with a medication regimen of ACTH, oral prednisolone, levetiracetam, sodium valproate, and clonazepam. When the oral prednisolone was discontinued for 3 months, recurrent seizures presented as eyelid blinking when nervous, which was considered eating-induced. When prednisone was administered, he did not have episodes of seizure for more than a month until the last follow-up. Patient 4 had been seizure-free for 10 months on medication containing sodium valproate, levetiracetam, and nitrazepam. The remaining three patients still suffered from seizures of varying frequency and severity despite being given continuous treatment with multiple anti-seizure medications. Patient 5 took the valproate first, and the frequency was cut down to half. However, 5 days later, he suffered more frequent seizures. When the levetiracetam was subscribed, the same reaction process occurred. He was intolerant of the nitrazepam, topiramate, and perampanel. Then, the lacosamide was added to his medication regime. It worked to reduce the frequency, but he still suffered seizures every day. For patient 6, the oxcarbazepine was first given and the seizures became more frequent. Then, she took sodium valproate. Instead, the seizures were reduced. Then, levetiracetam and nitrazepam were added. The drugs worked at first, but then her seizures became drug-resistant. Then, she tried clobazam and lamotrigine, and ketogenic diet therapy (KDT). The seizure frequency was halved after 1 month of KDT. Patient 7 took sodium valproate firstly with the seizure frequency reduced. Then, the levetiracetam and nitrazepam were added, and his seizure was reduced gradually. Although the KDT was effective in epilepsy and could benefit mental development, the efficacy of KDT for patients with *SYNGAP1* gene mutation was not previously reported.

## Discussion

*SYNGAP1* encephalopathy is a genetically determined brain disease and a significant cause of NDD and developmental and epileptic encephalopathy (DEE) in children (Vlaskamp et al., 2019). It is an attractive candidate for in-depth research across multiple model systems. *SYNGAP1* gene has become a high-risk gene for neuropsychiatric disorders in the differential diagnosis of NDD (Kilinc et al., 2018). In this study, we described phenotypes in patients with *SYNGAP1* gene mutations, which were characterized by intellectual disability, epilepsy, ASD, and behavioral problems. Epileptic spasms were captured in three of the seven patients probably because our patients underwent video-EEG examinations during infancy.

The global developmental delay was the first manifestation in patients with *SYNGAP1* gene mutations associated encephalopathies of this study. Intellectual or developmental disabilities (IDDs) appeared within the first year of life and deteriorated with age. In addition, the language impairment was more severe than the motor delay, which is similar to what has been reported in two previously reported cohorts (Parker et al., 2015; Mignot et al., 2016). Moreover, little improvements were observed in all patients after specific training, particularly for those with language impairment. However, the information about the long-term follow-up about their adult living ability was still lacking.

The most prominent clinical feature among seizure types in our patient cohort was myoclonic seizures, which was consistent with previously published literature (Parker et al., 2015; Mignot et al., 2016). The main EEG features in our patients were generalized bursts of spikes, poly spikes, spikes, and slow waves, sometimes with an occipital predominance. While Vlaskamp et al. first reported spasms in 1 of 57 children, we found that 42.8% (three of seven) of our patients suffered epileptic spams. In this study, fewer children were induced by eating (von Stulpnagel et al., 2019), and the number of children with aggressive behavioral problems combined with ASD was also lower than that reported in the literature (Weldon et al., 2018).

Taking into account the six patients reported, all the patients experienced a developmental delay. Moreover, according to the limited information, we could learn that mostly moderate developmental delay was observed among patients; 84.6% (11 out of 13) of children experienced seizures, similar to the previous study (Parker et al., 2015; Mignot et al., 2016).

It was consistent with the literature (Vlaskamp et al., 2019). Most of the mutations identified in the present study were *de novo* heterozygous mutations that caused losses of function of SynGAP due to conformational defects of frameshifted, splice-site, and truncated mutations. Only one study had so far reported mutations in exon 1 (Tian et al., 2020), which caused the loss of promoters for the transcription. However, the mutation frequency of the *SYNGAP1* gene in the Chinese population was unknown due to the lack of a large multicenter prospective cohort study. Thus, the exact function in the pathogenesis of the disease remains uncertain. Previous studies reported that mutations in *SYNGAP1* gene caused NDD by inducing haploinsufficiency (Berryer et al., 2013). Animal models suggested that homozygous SynGAP-/- mice were not able to survive after 48 h (Berryer et al., 2013). Based on the results from an animal experiment, different mutation subtypes have different effects on neuronal activity (Araki et al., 2020), while how different gene mutations, such as mutation forms of mutation loci, influence phenotypes and prognosis remained unknown (Agarwal et al., 2019). However, we could not distinguish the difference in the clinical features among different domains.

At the last follow-up, three children had recurrent seizures and poor responses to the anti-seizure medications. Four children never achieved seizure-free effect. All the patients were taking three or more anti-seizure medications. All patients suffered from drug-resistance epilepsy, which was higher than what has been reported in a large cohort of 57 patients with *SYNGAP1*-DEE (Vlaskamp et al., 2019). It has been reported that epilepsy induced by *SYNGAP1* gene mutations responded better to topiramate and cannabidiol (Kuchenbuch et al., 2020), and we found that valproate seemed of benefit to our patients. There was no related efficacy of the ketogenic diet in children with *SYNGAP1* mutation reported in the literature. We need more experience about the effect of KDT on seizures and development improvement in *SYNGAP1* gene-related patients.

This study has several limitations. This study is an observational retrospective study of a relatively small cohort of patients, and hence, the actual frequency of the *SYNGAP1* gene mutation in children is unknown. Abnormal EEG patterns were observed in five patients before the onset of seizures. Whether the prompt treatment for a patient with an abnormal EEG can prevent development plateauing or regression, even to improve growth and cognitive development, requires further investigation. Additionally, the limited age range and follow-up time window of the patients could not provide the long-term prognosis. Ample multicenter research with long-term follow-up is still needed to see how mutation forms of mutation loci relate to phenotypes and prognosis.

## Conclusion

Developmental delay and epilepsy are the main clinical manifestations in patients with *SYNGAP1* gene mutations. *SYNGAP1* gene variants are mainly *de novo*. Almost all patients showed varying degrees of intellectual deficiency and developmental delay before the onset of epilepsy. The primary seizure type in *SYNGAP1*-related epilepsy was myoclonic seizures. Abnormal EEGs were observed in most patients, and we predicted that the earlier administration of ASMs might be beneficial to the patients due to the fact that epilepsy was common among these patients and some experience developmental regression after the onset of seizures. We had not found the genotype–phenotype correlation, and the severity of disease correlated with the level of the protein truncation.

## Data Availability Statement

The original contributions presented in the study are included in the article/Supplementary Material, further inquiries can be directed to the corresponding author.

## Ethics Statement

The studies involving human participants were reviewed and approved by the Shenzhen Children’s Hospital. Written informed consent to participate in this study was provided by the participants’ legal guardian/next of kin.

## Author Contributions

HZ, LY, and JD designed the study. JL obtained funding. JH, YF, QZ, and HZ acquired the data. HZ, LC, DC, and JL analyzed clinical records and interpreted the data. HZ and JL drafted and revised the manuscript. All authors revised this draft, read, and approved the final manuscript.

## Conflict of Interest

The authors declare that the research was conducted in the absence of any commercial or financial relationships that could be construed as a potential conflict of interest.

## Publisher’s Note

All claims expressed in this article are solely those of the authors and do not necessarily represent those of their affiliated organizations, or those of the publisher, the editors and the reviewers. Any product that may be evaluated in this article, or claim that may be made by its manufacturer, is not guaranteed or endorsed by the publisher.

## Abbreviations

ACMG: American College of Medical Genetics and Genomics
ASMs: anti-seizure medications
ASD: autism spectrum disorder
CT: computed tomography
DEE: developmental and epileptic encephalopathy
EEG: electroencephalogram
GTPase: guanosine triphosphatase
ID: intellectual disability
KDT: ketogenic diet therapy
MRI: magnetic resonance imaging
MRD5: mental retardation type 5
NDD: neurodevelopmental disorders
SD: standard deviation
SynGAP: synaptic guanosine triphosphatase activating protein
*SYNGAP1*: synaptic Ras GTPase activating protein 1
WES: whole-exome sequencing.
