# Supplementary figures and images for: Phenotypes in Children With SYNGAP1 Encephalopathy in China
Source: Front Neurosci. 2021 Dec 2;15:761473. doi: 10.3389/fnins.2021.761473 (PMC8678593; doi:10.3389/fnins.2021.761473)

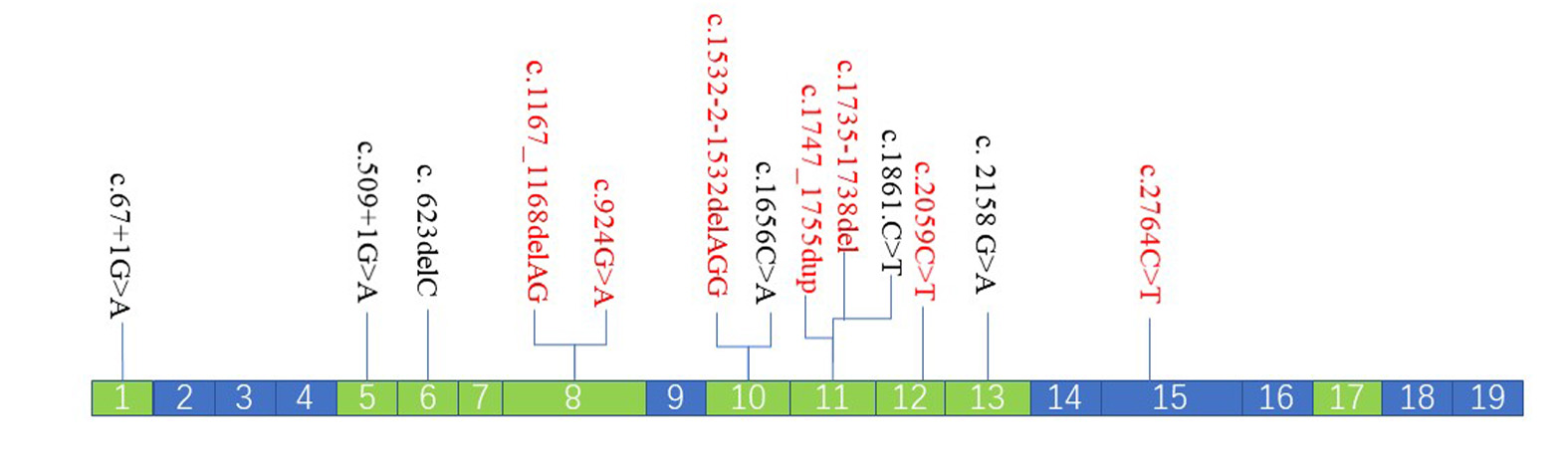

Supplement: Supplementary file 1 [file Image_1.jpg]
